# Nanotech meets antibiotics: nucleotide antibiotics delivered by lipid nanoparticles

**DOI:** 10.3389/fcimb.2025.1737088

**Published:** 2026-02-23

**Authors:** Bookun Kim, Dajeong Kim, Choong-Min Ryu

**Affiliations:** 1 Molecular Phytobacteriology Laboratory, Infectious Disease Research Center, Korea Research Institute of Bioscience and Biotechnology (KRIBB), Daejeon, Republic of Korea; 2 Department of Biosystems and Bioengineering, Korea Research Institute of Bioscience and Biotechnology (KRIBB) School of Biotechnology, University of Science and Technology, Daejeon, Republic of Korea

**Keywords:** antimicrobial resistant bacteria, lipid nanoparticles (LNPs), microfluidics, nucleotide delivery, nucleotide antibiotics, bacteria-targeting lipid nanoparticles

## Abstract

The global spread of multidrug-resistant bacteria poses a serious challenge to effective therapy and public health. The rising resistance to small-molecule antibiotics underscores the limitations of conventional antimicrobial strategies and highlights the urgent need for alternative therapeutic modalities. Lipid nanoparticles (LNPs) have emerged as efficient vehicles for delivering genetic materials, as exemplified by their success in mRNA vaccines. Recent studies suggest that LNPs can also be harnessed to suppress bacterial proliferation and counteract antibiotic resistance through the targeted delivery of nucleic acid cargo. In this review, we discuss recent advances in nanotechnology-based platforms for nucleic acid delivery into prokaryotic systems, with a particular focus on LNPs. We highlight LNPs as a promising delivery system for modulating antimicrobial resistance and bacterial fitness genes. Additionally, we outline key components and formulation strategies required to enable effective nucleic acid delivery against multidrug-resistant bacteria.

## 1 Introduction

During the coronavirus disease 2019 (COVID-19) pandemic, over-prescription and misuse of antibiotics became widespread, largely due to patients’ susceptibility to secondary infections and diagnostic uncertainty (Hsu, 2020). This excessive use of antibiotics during the pandemic is belived to have accelerated the emergence and dissemination of antimicrobial-resistant (AMR) bacteria (Rawson et al., 2020; Pelfrene et al., 2021). Indeed, antibiotic resistance has been described as a “silent pandemic” in itself (Mahoney et al., 2021). Although new antibiotics have been developed over recent decades to combat AMR pathogens, the rate at which resistance emerges has consistently outpaced the development of new antimicrobial agents (Årdal et al., 2020). The growing prevalence of antibiotic resistance thus represents one of the most pressing challenges in modern medicine (Christaki et al., 2020).

**Figure 1:**
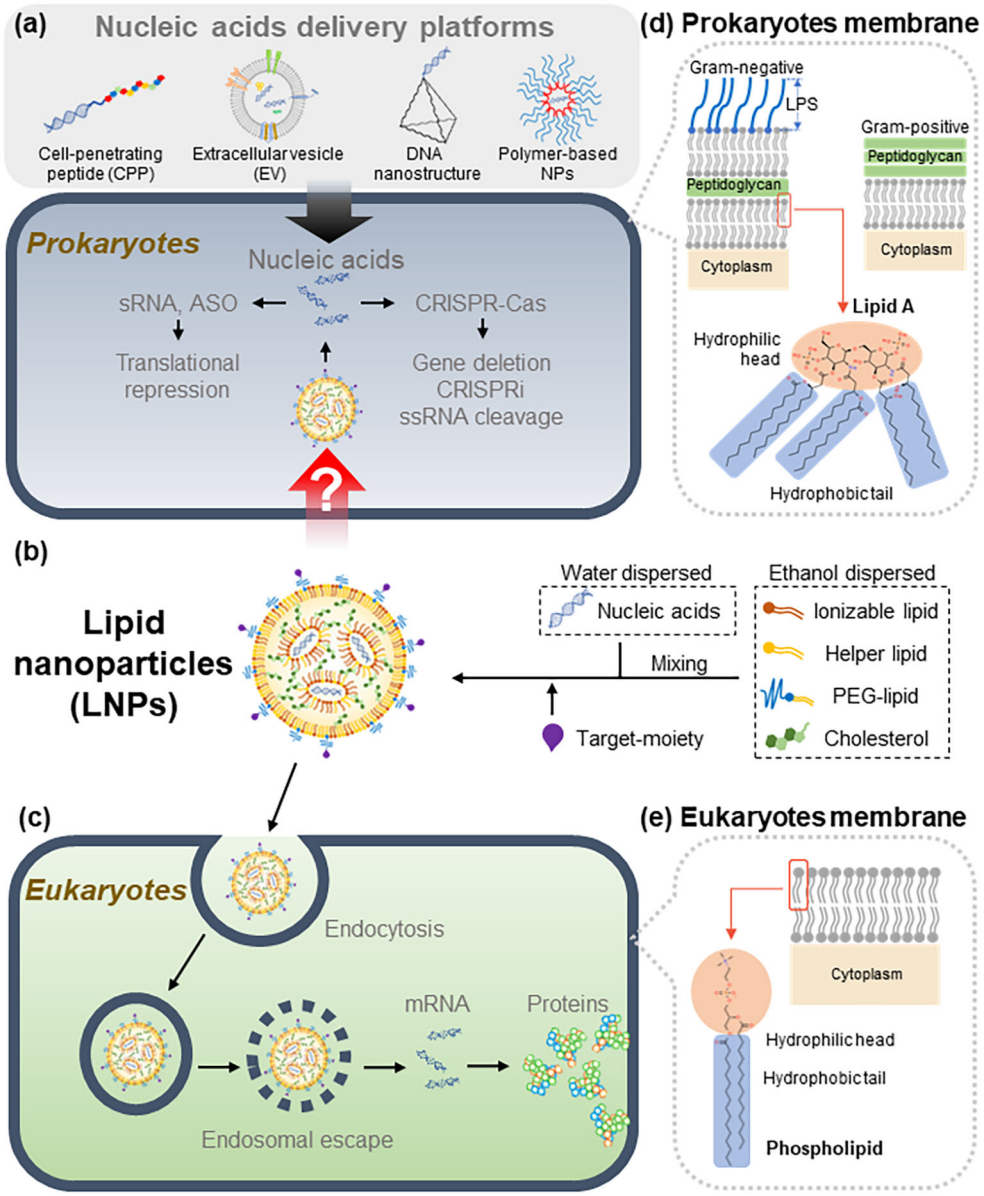
LNP-mediated delivery of nucleic acids into prokaryotic and eukaryotic cells. **(a)** In prokaryotes, nucleic acids were delivered by previous delivery platforms such as cell-penetrating peptide (CPP), extracellular vesicle (EV), DNA nanostructure, and polymer-based nanoparticles (NPs). **(b)** Schematic representation of LNPs as delivery system of nucleic acids. Components of LNPs are ionizable lipids, helper lipids, cholesterol, PEG-lipids (dispersed in ethanol) and nucleic acids (dispersed in water). When the water and ethanol solutions are mixed, LNPs are produced. Target- moiety can be attached to the surface of LNPs. LNPs pass through the bacterial membrane via an unknown mechanism. Released nucleic acids such as mRNAs, ASOs, and CRISPR-Cas systems then regulate the expression of target genes. **(c)** In eukaryotes, LNPs are captured by endocytosis. Due to the acidic environment of the endosome, nucleic acids are released into the cytosol (endosomal escape). In the case of mRNA vaccines, released mRNA is translated into proteins, leading to the activation of immune responses. **(d)** Structural differences between Gram-negative and Gram-positive bacterial membranes. Gram-negative bacteria have an inner membrane and an outer membrane, with a peptidoglycan layer between them. The outer membrane had a lipopolysaccharide (LPS) layer. Gram-positive bacteria had a single membrane and a thick layer of peptidoglycan. **(e)** Eukaryotic cells have a single membrane without a peptidoglycan layer, which is made of phospholipids. LNPs, lipid nanoparticles; CPP, cell-penetrating peptide; EV, extracellular vesicle; NPs, nanoparticles; sRNA, small RNA; ASO, antisense oligonucleotide; CRISPRi, CRISPR interference; ssRNA, single-stranded RNA. The chemical structure of each lipid was different (lipid A, phospholipid).

To address this global threat, alternative therapeutic strategies have been explored, including the use of nucleic acid–based agents. Delivery platforms such as cell-penetrating peptides (CPPs), extracellular vesicles (EVs), DNA nanostructure, and polymer-based nanoparticles (NPs) have been actively investigated as potential solutions (**Figure 1a**) (Xue et al., 2018; Moreira et al., 2024). Such platforms aim to overcome biological barriers and improve the intracellular delivery efficiency of therapeutic nucleic acids. However, achieving efficient and safe delivery of these molecules to target sites remains a formidable challenge (Wojciechowska et al., 2020; Bier and Nizet, 2021).

Nucleotide-based antibiotics represent a new class of programmable therapeutics that employ nucleic acids—such as antisense oligonucleotides, small RNAs, or CRISPR–Cas systems—to silence essential genes, inhibit virulence pathways, or eliminate antimicrobial resistance determinants in bacteria (Wan et al., 2021; Moreira et al., 2024). Unlike conventional small-molecule antibiotics, these agents act through sequence-specific base pairing or targeted gene editing, enabling highly precise bacterial gene modulation. However, their clinical potential has been limited by the absence of an efficient delivery system capable of transporting nucleic acids across the complex bacterial cell envelope. In this context, lipid nanoparticles (LNPs) offer a uniquely suitable platform: their modular lipid architecture enables encapsulation, protection, and controlled release of nucleic acid cargo (**Figure 1b**) (Cullis and Felgner, 2024), providing the essential technological bridge that links nucleotide-based antibiotics to practical antimicrobial applications. Thus, the development of LNPs directly underpins the feasibility and therapeutic utility of nucleotide antibiotics.

The delivery platform is classified into two types: encapsulation and transporter. In the case of encapsulation, nucleic acids are encapsulated in materials that can enter the cytosol, where nucleic acids are released [e.g., EVs, polymer-based NPs, LNPs]. Nucleic acids can be linked to transporter materials that can penetrate the bacterial membrane, carrying the cargo into the target cell [e.g., CPPs and DNA nanostructures] (**Table 1**).

**Table 1:** Types of nucleic acid delivery nano-systems.

Type		Feature
Encapsulation: Nucleic acids are encapsulated in materials that can enter the cytosol, where nucleic acids are degraded to release the nucleic acid cargo.	Lipid nanoparticles (LNP) 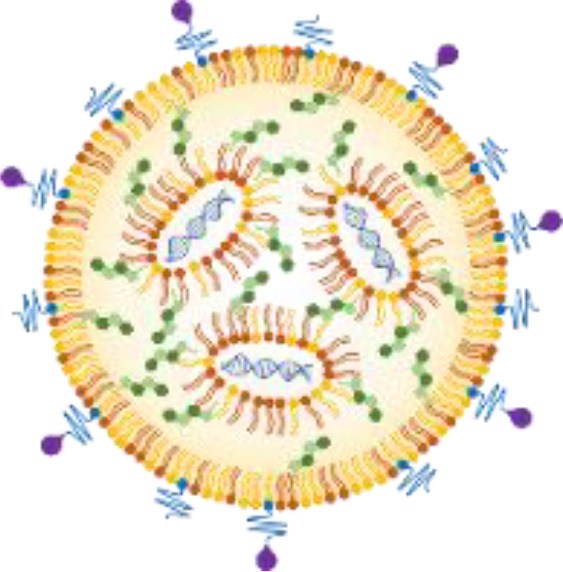	Nucleic acids are encapsulated by artificially synthesized organic lipids and cholesterol through a microfluidic mixing technique. Targeting moieties can also be conjugated to the particle surface. LNPs enable large-scale production at low cost and were successfully used as vaccine platforms during the COVID-19 pandemic. *Limitations: prone to immune responses and stability issues
	Polymer-based nanoparticles 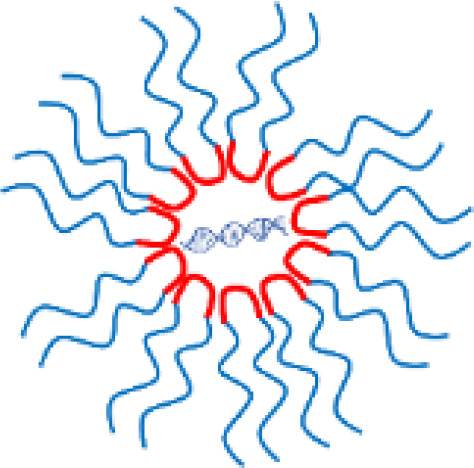	Micelles and dendrimers are formed via electrostatic self-assembly between polymers and nucleic acids. The synthetic polymeric structures encapsulate nucleic acids and protect them from nuclease degradation. *Limitations: potential toxicity, complex manufacturing, and possible immune responses
	Extracellular vesicles (EVs) 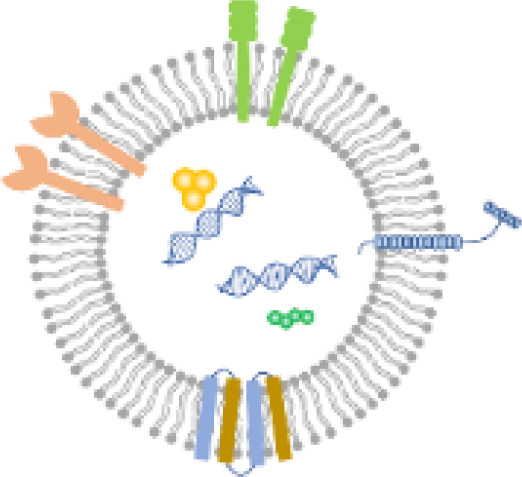	Lipid-bound vesicles naturally secreted by cells into the extracellular environment. Intracellular nucleic acids are enclosed by the cell membrane prior to release. *Limitations: low production yield, challenges in standardization, and complex purification processes
Transporter: Nucleic acids are linked to materials (transporters) that can penetrate the bacterial membrane, carrying the cargo into the target cell.	DNA nanostructures 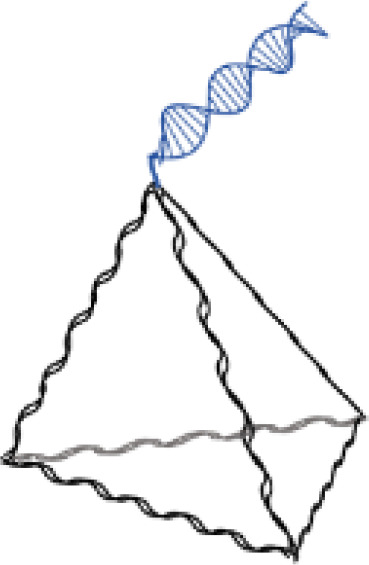	Nanoscale architectures composed of DNA that can be engineered as delivery platforms. *Limitations: susceptible to rapid degradation, high cost, and potential immune activation
	Cell-penetrating peptides (CPPs) 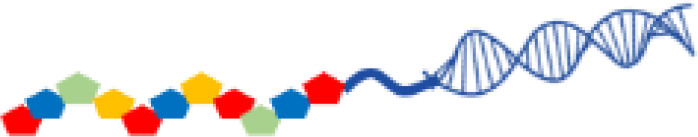	Short peptides (<30 amino acids) capable of crossing bacterial membranes and transporting conjugated molecules into cells. *Limitations*: risk of hemolysis, nonspecific delivery, and stability concerns

EVs are lipid-bound vesicles secreted by cells into the extracellular surroundings. Intracellular nucleic acids are wrapped in the cell membrane before release. EVs are produced by encapsulation in the membranes of bacterial cells. Thus, the target cell membrane’s surface selectivity and lipid compatibility represent advantages. The decisive drawback is the difficulty in isolating EVs from cells. Ultracentrifugation and isolation were required to separate microbial EVs. EVs were lost during the isolation process, which reduced production efficiency (Onódi et al., 2018). For this reason, EVs were still being used at the laboratory scale (Bost et al., 2021).

Polymer-based NPs (micelles and dendrimers) are produced by the electrostatic self-assembly of polymers and nucleic acids. NPs can alter the properties of nanoparticles by modifying the molecular structure of the polymer shell (size, surface properties) (Makino et al., 2014). Size control was an advantage compared with EVs, but the control range was narrow (diameter 10−100 nm) (Tapeinos et al., 2017). The use of organic solvents for the preparation of nanoparticles was another disadvantage that can increase toxicity (Tapeinos et al., 2017).

CPPs are characterized by their ability to penetrate the bacterial membrane. CPPs were used as nucleic acid carriers, taking advantage of their membrane-penetrating properties (Xue et al., 2018). CPPs effectively delivered nucleic acids into the bacterial inner membrane under *in vitro* conditions (Wojciechowska et al., 2020). However, under *in vivo* conditions, practical applications were not possible due to the side effect of hemolysis (Wojciechowska et al., 2020).

DNA nanostructures are combinations of complementary gene sequences assembled into 2D and 3D nano-transporters (tetrahedron, octahedron, cube, etc.) (Xue et al., 2018). Nucleic acids can be delivered to bacteria in the form of nanostructures-linker-nucleic acids (Wojciechowska et al., 2020). DNA nanostructures are ideal transporters with many advantages. However, it was still only possible to produce them at the laboratory scale, and the unit price was unpredictable (Xue et al., 2018; Wojciechowska et al., 2020).

LNPs are fatty particles used to deliver therapeutic molecules like mRNA by protecting them from degradation and helping them enter eukaryotic cells (**Figure 1c**). LNPs have demonstrated remarkable promise as delivery vehicles, most notably during the COVID-19 pandemic, when mRNA encapsulated in LNPs was successfully utilized in vaccines. The benefits of LNP-based mRNA vaccines—such as stability, scalability, rapid development, and economic feasibility—have been well documented (Meo et al., 2021). Notably, the pivotal role of LNPs in mRNA technology was recognized through consecutive Nobel Prizes in Physiology or Medicine in 2023 and 2024, underscoring their significance as a transformative platform for future gene delivery (Karikó et al., 2005; Ambros and Ruvkun, 2018).

Building on these advancements, we established a foundation for exploring the potential application of LNPs in targeting bacterial systems (Kim et al., 2025). In this review, we propose novel avenues for applying LNPs to the treatment of AMR bacteria. By integrating physicochemical and biological design strategies, our aim is to optimize LNP formulations for efficient and targeted delivery to resistant bacterial strains. Ultimately, this approach seeks to enhance the therapeutic efficacy of nucleic acid–based agents and contribute to innovative solutions addressing the escalating challenge of antibiotic resistance. Continued investigation in this field may pave the way for next-generation antimicrobial therapies that safeguard global health.

## 2 Overview of LNPs for nucleotide-based antimicrobials

LNPs have emerged as one of the most versatile and clinically validated platforms for nucleic acid delivery, as evidenced by their widespread use in mRNA vaccines and gene therapy applications (Swingle et al., 2021; Barbier et al., 2022). Their modular composition, physicochemical tunability, and manufacturing scalability position LNPs as a promising foundation for the development of nucleotide-based antimicrobial agents. In the context of bacterial systems, where membrane architecture and permeability significantly differ from those of eukaryotic cells, understanding the structural features and delivery mechanisms of LNPs is essential for rational design. This section provides an overview of the fundamental components of LNPs, the mechanistic basis of nucleic acid encapsulation and delivery, and the intrinsic advantages that make LNPs strong candidates for next-generation nucleotide antibiotics.

### 2.1 Composition and physicochemical characteristics

LNPs are self-assembled nanostructures composed of ionizable lipids, helper lipids, cholesterol, and polyethylene glycol (PEG)–conjugated lipids, typically formed through rapid microfluidic mixing of an ethanol phase containing lipids with an aqueous acidic phase containing nucleic acids (**Figure 1b**) (Cullis and Felgner, 2024). Each component plays a distinct structural and functional role:

Ionizable lipids form the core of the nanoparticle. They are positively charged under acidic conditions, facilitating electrostatic complexation with negatively charged nucleic acids. At physiological pH, they become neutral, thereby minimizing cytotoxicity and nonspecific interactions (Schlich et al., 2021). Their molecular structure—including head-group pKa, linker flexibility, and tail hydrophobicity—critically influences membrane fusion and the release of nucleic acid cargo (Cullis and Felgner, 2024).

Helper lipids, such as phospholipids, contribute to membrane fluidity and support fusion between LNP surfaces and cellular membranes (Albertsen et al., 2022). These lipids enhance the structural organization of the particle and aid in stabilizing the lipid bilayer during encapsulation and delivery (Jia et al., 2024).

PEG–lipids provide steric stabilization of the particle surface, preventing aggregation during formulation and storage (Jia et al., 2024). PEGylation also prolongs circulation time *in vivo*, although excessive PEG density may reduce cellular uptake, necessitating careful optimization (Jia et al., 2024).

Cholesterol improves the mechanical rigidity and stability of LNPs (Albertsen et al., 2022). It also facilitates lipid packing, reduces membrane permeability, and modulates the overall shape and fluidity of the nanoparticle, which are essential for efficient intracellular transport (Albertsen et al., 2022; Jia et al., 2024).

These modular components allow customizable tuning of surface charge, lipid packing, hydrophobicity, and particle size—parameters that directly affect the interaction of LNPs with bacterial membranes. As demonstrated in studies of LNP–eukaryotic membrane interactions (Schlich et al., 2021; Cullis and Felgner, 2024), these physicochemical parameters are likewise expected to play critical roles in governing how LNPs engage with bacterial cell envelopes.

### 2.2 Mechanistic basis for nucleic acid encapsulation and delivery

LNPs are formed through rapid microfluidic mixing, during which ionizable lipids electrostatically complex with nucleic acids and self-assemble into nanoparticles with tunable diameters typically ranging from 50–150 nm (Maeki et al., 2015; Shepherd et al., 2021). For delivery into bacteria, LNPs must traverse cell envelopes that differ substantially from eukaryotic membranes: Gram-negative bacteria possess an outer membrane enriched with lipopolysaccharides (LPS), while Gram-positive bacteria have a thick peptidoglycan layer, both of which act as formidable permeability barriers (**Figure 1d**). Although LNPs were originally optimized for endosomal escape in eukaryotic systems (Schlich et al., 2021), how LNPs overcome the structural barriers of bacterial membranes is still poorly understood. Recent evidence suggests that delivery efficiency can be enhanced through the use of LNP-helper molecules such as polymyxins, which transiently disrupt LPS structure and facilitate nanoparticle penetration (Kim et al., 2025). After membrane perturbation, the encapsulated nucleic acids—such as CRISPR–Cas systems—may be released into the bacterial cytosol, where they modulate gene expression or induce targeted killing (Kim et al., 2025). While the precise molecular pathways underlying these processes remain to be fully elucidated, current studies collectively indicate that the physicochemical properties of LNPs govern their ability to overcome bacterial envelope barriers and achieve intracellular delivery.

### 2.3 Advantages of LNPs as nucleotide antibiotics

**Figure 2:**
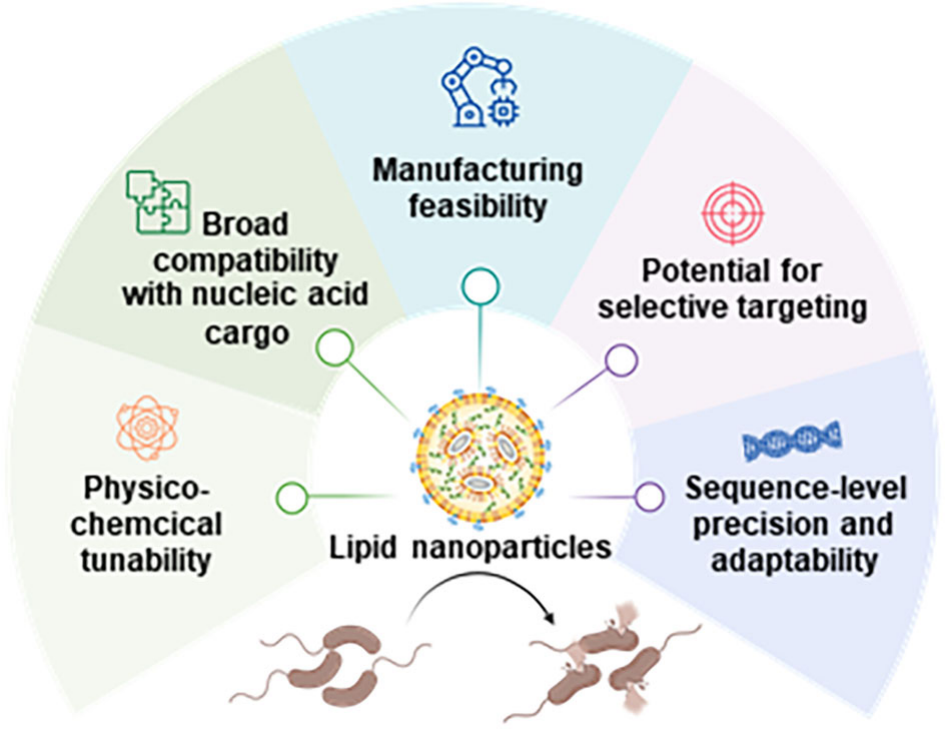
Schematic illustration highlighting the key advantages of LNPs as a delivery platform for nucleotide antibiotics. LNPs offer (1) physicochemical tunability, enabling precise control over particle size, surface charge, and lipid composition; (2) broad compatibility with diverse nucleic acid cargos, including CRISPR–Cas systems; (3) manufacturing feasibility through scalable and reproducible microfluidic production; (4) potential for selective targeting via surface modification with targeting moieties; and (5) sequence-level precision and adaptability, allowing rapid reprogramming to counter emerging antimicrobial resistance.

The structural versatility and physicochemical customizability of LNPs underpin several key advantages for the development of nucleotide-based antimicrobial strategies (**Figure 2**). Importantly, these advantages arise directly from the modular molecular design of LNPs.

#### 2.3.1 Physicochemical tunability

The synthetic nature of LNP lipids enables fine control over particle size, surface charge, hydrophobicity, and fusogenicity (ref). Adjusting head-group pKa or hydrophobic tail structure allows researchers to engineer LNPs capable of interacting with the lipid architectures of eukaryotic cell membranes (ref). This tunability can also provide a rational path to overcoming the unique permeability barriers of Gram-negative and Gram-positive bacteria.

#### 2.3.2 Broad compatibility with nucleic acid cargo

Unlike other delivery systems, LNPs can encapsulate a wide range of nucleic acids—including mRNA, antisense oligonucleotides, plasmid DNA, and CRISPR–Cas components—without extensive redesign (Chaudhary et al., 2021). This flexibility is particularly advantageous for antibacterial applications, where the optimal molecular modality may differ by target gene, resistance mechanism, or bacterial species.

#### 2.3.3 Manufacturing feasibility

Microfluidic production enables highly reproducible generation of LNPs at laboratory and industrial scales (Maeki et al., 2018). This property is essential for antimicrobial development, where rapid formulation optimization and scalable production are prerequisites for therapeutic translation.

#### 2.3.4 Potential for selective targeting

Functional moieties can be conjugated to the LNP surface to enhance target specificity and delivery efficiency (Li et al., 2018; Chaudhary et al., 2021). For example, PEG–lipid anchors allow for the conjugation of targeting moieties—including antibodies, peptides, and enzymatic ligands—that can selectively bind to bacterial surface structures (**Figure 1b**). This feature offers a path toward reducing off-target effects and enhancing delivery efficiency in complex microbiome environments.

#### 2.3.5 Sequence-level precision and adaptability

Because nucleic acid cargo can be reprogrammed rapidly in response to newly emerging resistance determinants, LNP-based nucleotide antibiotics offer exceptional adaptability. The adaptability through rapid reprogramming can establish LNP-based nucleotide antibiotics as a sustainable and continuously adaptable strategy for countering the ongoing emergence of antimicrobial resistance.

Collectively, these advantages highlight LNPs as a powerful and adaptable platform for delivering nucleic acid therapeutics into bacterial cells. The unique combination of mechanical stability, manufacturability, and molecular precision positions LNPs as a leading candidate for next-generation antimicrobial interventions.

## 3 Design strategies of nucleotides as antibiotics

To effectively control bacterial pathogens, nucleic acids must be designed to target specific bacterial genes. These targets can be broadly classified into three categories: essential genes, virulence factors, and AMR genes. The first strategy involves inhibiting essential genes required for vital cellular processes such as cell wall synthesis, DNA replication, RNA transcription, and protein translation. Silencing these genes leads to bacterial death. However, such broad-spectrum antibacterial activity may also eliminate beneficial members of the human microbiota. To overcome this limitation, anti-virulence therapies have been proposed (Clatworthy et al., 2007). A more refined approach is to suppress bacterial virulence factors that enable pathogens to colonize, invade, and persist within host tissues. Nucleic acid–based strategies have been designed to interfere with biofilm formation, toxin–antitoxin systems, and quorum sensing networks (Da et al., 2017; Równicki et al., 2018; Zhang et al., 2020). The third approach focuses on targeting AMR genes. Rather than killing bacteria directly, gene-editing technologies such as the clustered regularly interspaced short palindromic repeats–CRISPR-associated protein (CRISPR–Cas) system can disable resistance determinants, thereby restoring bacterial susceptibility to antibiotics (Kang et al., 2017; Wan et al., 2021).

**Figure 3:**
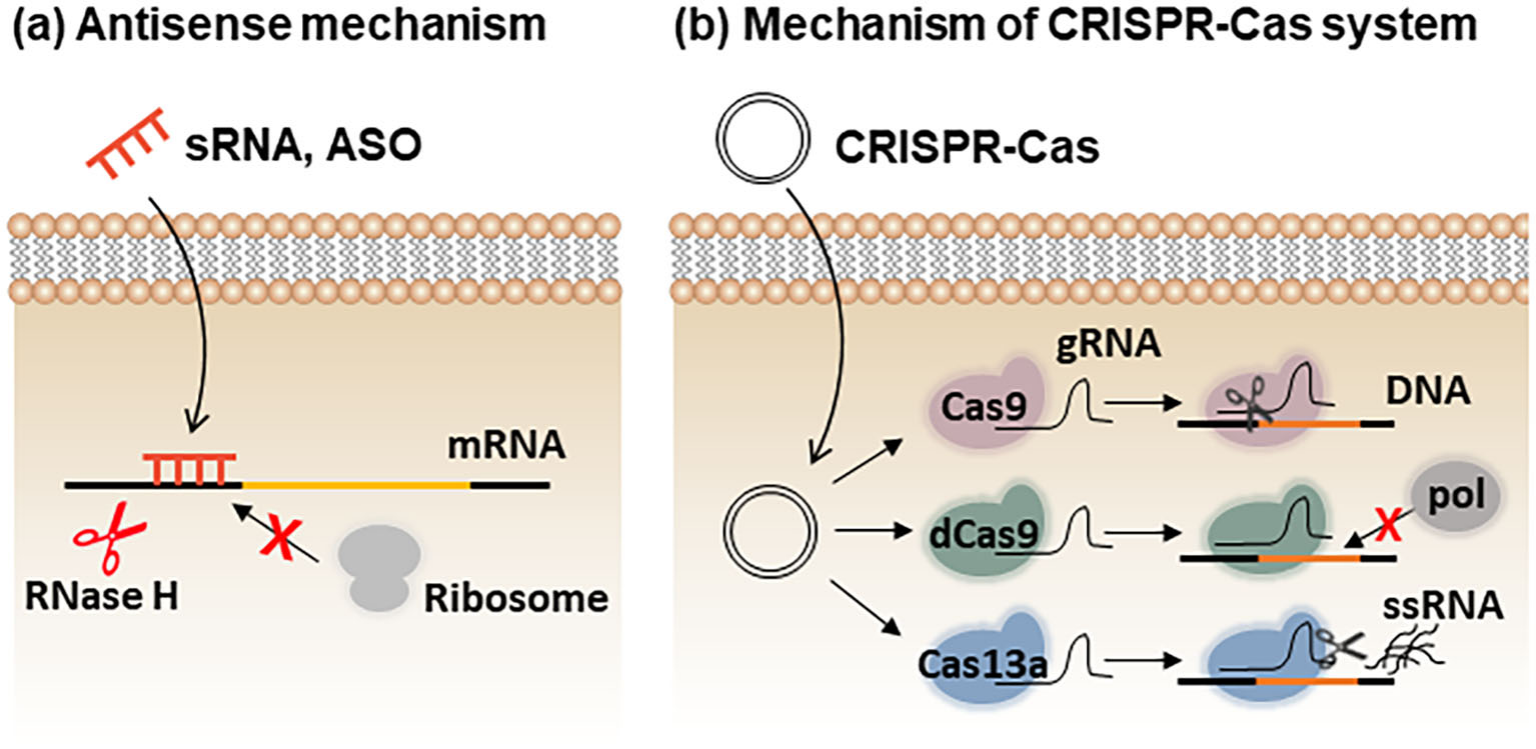
Types of nucleic acids and their gene regulation. **(a)** Antisense small RNAs (sRNAs) and antisense oligonucleotides (ASOs) hybridize with target mRNAs to form double-stranded complexes. These duplexes are recognized and degraded by RNase H, or alternatively, prevent ribosome binding, thereby suppressing target gene expression. **(b)** In CRISPR–Cas systems, the expressed Cas protein forms a complex with guide RNA (gRNA) to recognize the target sequence. Wild-type Cas9 cleaves target DNA, whereas catalytically inactive Cas9 (dCas9) binds to the target site and sterically blocks RNA polymerase binding. Cas13a recognizes target RNA sequences and cleaves surrounding non-specific single-stranded RNAs (ssRNAs).

The choice of nucleic acid type is also crucial. Regulatory small RNAs (sRNAs) can modulate gene expression post-transcriptionally by base-pairing with target mRNAs through antisense interactions (Lalaouna et al., 2013; Hegarty and Stewart, 2018). This pairing either blocks ribosome access to the ribosome-binding site (RBS) or triggers degradation of the sRNA–mRNA duplex via RNase H, ultimately inhibiting translation of the target gene (**Figure 3a**). Alternatively, antisense oligonucleotides (ASOs), chemically modified DNA or RNA analogs, can achieve similar regulatory effects. Structural modifications such as phosphorothioate (PS), peptide nucleic acid (PNA), or locked nucleic acid (LNA) backbones enhance nuclease resistance and cellular stability during delivery into prokaryotic cells (Hegarty and Stewart, 2018; Xue et al., 2018). To suppress multidrug-resistant bacteria by gene disruption, CRISPR–Cas systems have been widely explored (**Figure 3b**). The bacterial RNA-guided endonuclease Cas9 cleaves genomic DNA at sequences complementary to the guide RNA (gRNA), resulting in targeted killing or removal of antibiotic resistance genes (Ran et al., 2013; Bier and Nizet, 2021). A catalytically inactive variant, dCas9, retains DNA-binding capability but lacks nuclease activity; it represses transcription by sterically blocking RNA polymerase at promoter regions; a mechanism known as CRISPR interference (CRISPRi) (Zuberi et al., 2017; Zhang et al., 2021a). Since many antimicrobial resistance (AMR) genes are plasmid-encoded, cleavage by Cas9 alone may be insufficient to eradicate bacteria carrying high-copy plasmids (Citorik et al., 2014). To address this, CRISPR–Cas13a has been introduced owing to its collateral single-stranded RNA (ssRNA) cleavage activity (**Figure 3b**) (Kiga et al., 2020). Once activated by recognition of AMR gene transcripts, Cas13a indiscriminately degrades surrounding RNAs, effectively killing bacteria that harbor resistance genes (**Figure 3b**).

## 4 Development strategies for bacteria-targeting LNPs

Potential challenges in developing bacteria-targeting LNPs arise from the fundamental structural differences between bacterial and eukaryotic cell membranes (**Figures 1d, e**). Unlike eukaryotic membranes, bacterial membranes exhibit unique compositions and architectures (Sun et al., 2022). Consequently, it remains uncertain whether LNPs originally designed for eukaryotic cells can effectively deliver cargo into the bacterial cytosol. In Gram-negative bacteria, the presence of lipopolysaccharides (LPS) on the outer membrane (**Figure 1d**) can physically hinder LNP permeation. Another major obstacle is the inherently low delivery efficiency associated with the double-layered membrane structure of these bacteria. The distinct organization of bacterial membranes is believed to influence their mechanical strength and elasticity, further complicating nanoparticle penetration (Jauffred et al., 2007; Sun et al., 2022). However, how LNPs interact with and modulate the mechanical properties of the bacterial double membrane remains poorly understood.

Additional barriers arise from the thick peptidoglycan layer, particularly in Gram-positive bacteria, where the cell wall is substantially thicker than that of Gram-negative species (**Figure 1d**). Beyond these physical impediments, challenges also stem from the chemical composition of LNP lipids themselves (Chaudhary et al., 2021). The *endosomal escape mechanism* (**Figure 1c**)—in which LNP lipids fuse with cellular membranes to release encapsulated nucleic acids—is governed by the molecular structures of ionizable and helper lipids (Csiszár et al., 2015; Bost et al., 2021; Chaudhary et al., 2021). Under acidic conditions, the fusogenic properties of these lipids become critical, enabling the release of nucleic acids through membrane fusion with the target bacterial envelope (Csiszár et al., 2015).

### 4.1 Development of lipid organic synthesis

**Figure 4:**
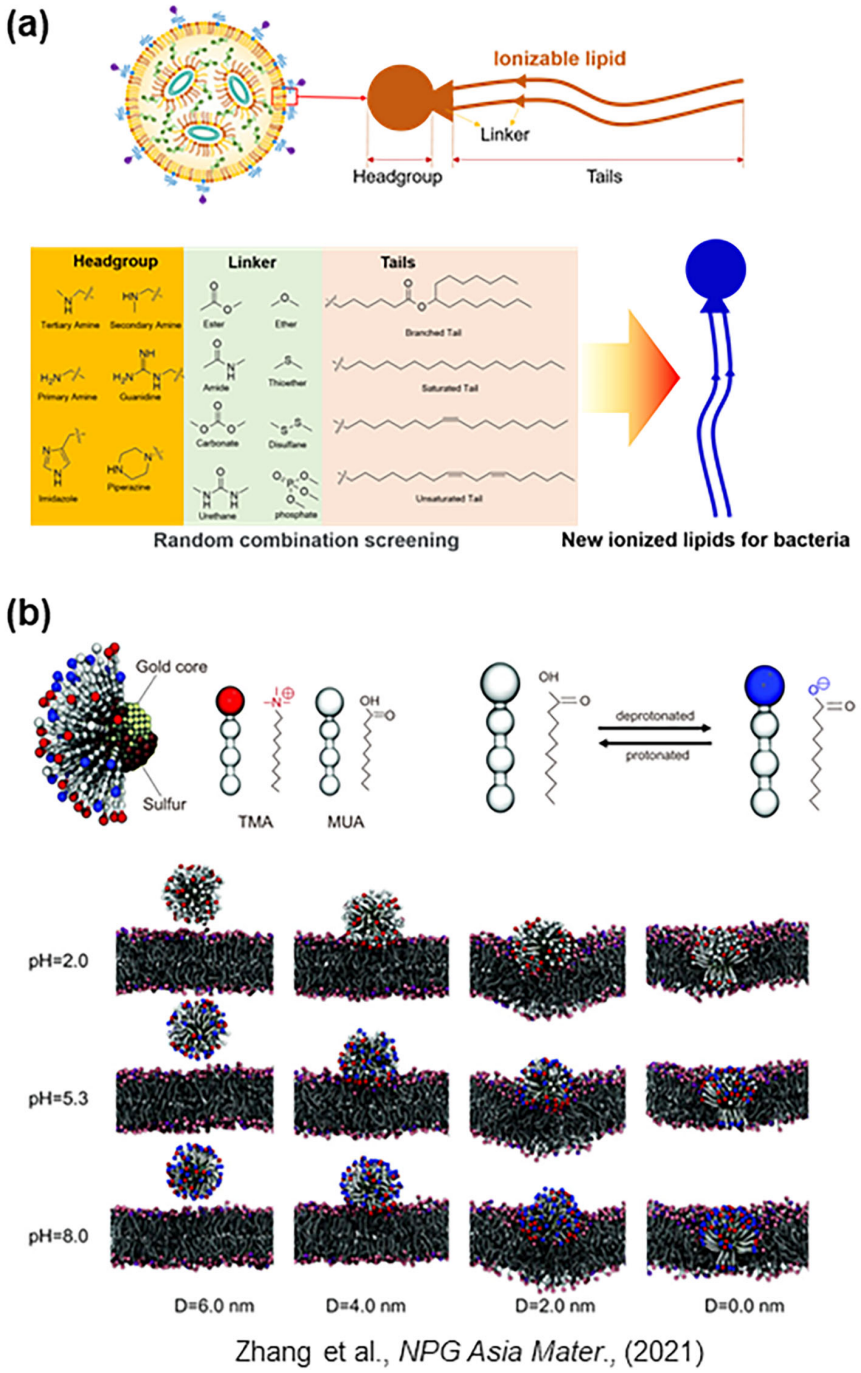
Development and optimization of LNPs. **(a)** Screening efforts for the development of ionizable lipids were conducted through random combinatorial synthesis of lipid head and tail structures to enhance fusion efficiency with cellular membranes. **(b)** Molecular dynamics (MD)–based computer simulations, such as those performed using the Large-scale Atomic/Molecular Massively Parallel Simulator (LAMMPS), were employed to model and predict the interaction of LNPs with lipid membranes, serving as an example of concurrent computational and experimental optimization.

The endosomal escape mechanism depends on the properties of lipids within LNPs. Therefore, the key milestone in the development of LNPs was the discovery of new lipids (Scheideler et al., 2020). The efficiency of nucleic acid delivery varies dramatically depending on the combination of the head (hydrophilic) and tail (hydrophobic) groups (**Figure 4a**) (Scheideler et al., 2020; Zhao et al., 2020; Dingjan and Futerman, 2021; Xu et al., 2023). It should be noted that the molecular structure of lipids differs between bacteria and eukaryotic cells (**Figures 1d, e**). For example, in *E. coli*, the membrane is composed of Lipid A, which has a hydrophilic head with two phosphate groups and six hydrophobic tails (**Figure 1d**) (Raetz et al., 2009). In contrast, lipids in eukaryote membranes possess a different structure, typically containing one phosphate head and two tails (Kotnik et al., 2012). Because the head-tail configurations of bacterial lipids differ from those of eukaryotic cell membranes, new combinations must be synthesized through organic chemistry steps. Generating numerous head-tail combinations is a laborious and time-consuming process, making it challenging to identify appropriate fusogenic lipids through simple experimental screening (Zhao et al., 2020; Dingjan and Futerman, 2021). To alleviate this effort, screening ionizable lipid structures in combination with computer simulations can be employed. Software such as GROMACS (Royal Institute of Technology, Sweden), LAMMPS (Sandia National Laboratories, USA), and NAMD (University of Illinois at Urbana-Champaign, USA) are useful tools for predicting how LNPs interact with membranes (**Figure 4b**) (Zhang et al., 2021b). The programs are based on molecular dynamics simulations.

### 4.2 Target moieties on LNPs

Attaching target moieties to the surface of LNPs can be considered a viable strategy for minimizing cytotoxicity and achieving selective nucleic acid delivery (**Figure 1b**). Reduced surface specificity can result in nonspecific cellular uptake of LNPs, thereby increasing cytotoxicity (Tapeinos et al., 2017; Scheideler et al., 2020). Conjugating appropriate target moieties can stabilize LNPs on bacterial surfaces, facilitate the overcoming of physical barriers, and promote the delivery of nucleic acids into the cytosol. Moreover, proteins account for approximately 70% of the bacterial surface, providing abundant sites for targeted interactions. Delivery efficiency can thus be enhanced by conjugating antibodies, ligands, peptides, or other protein-based moieties to the LNP surface (Mccrorie et al., 2020; Sun et al., 2022). For instance, endolysins as targeting moieties can assist LNPs in penetrating the peptidoglycan layer by anchoring to the bacterial cell wall (Love et al., 2020). While targeting moieties can significantly improve cellular uptake, it remains unclear whether they also influence endosomal escape efficiency. The mechanisms by which LNPs release their nucleic acid cargo after entry into bacterial cells are still not fully elucidated. Therefore, a multifaceted and systematic scientific approach will be essential for advancing the development of bacteria-targeting LNPs.

### 4.3 LNP-helper

**Figure 5:**
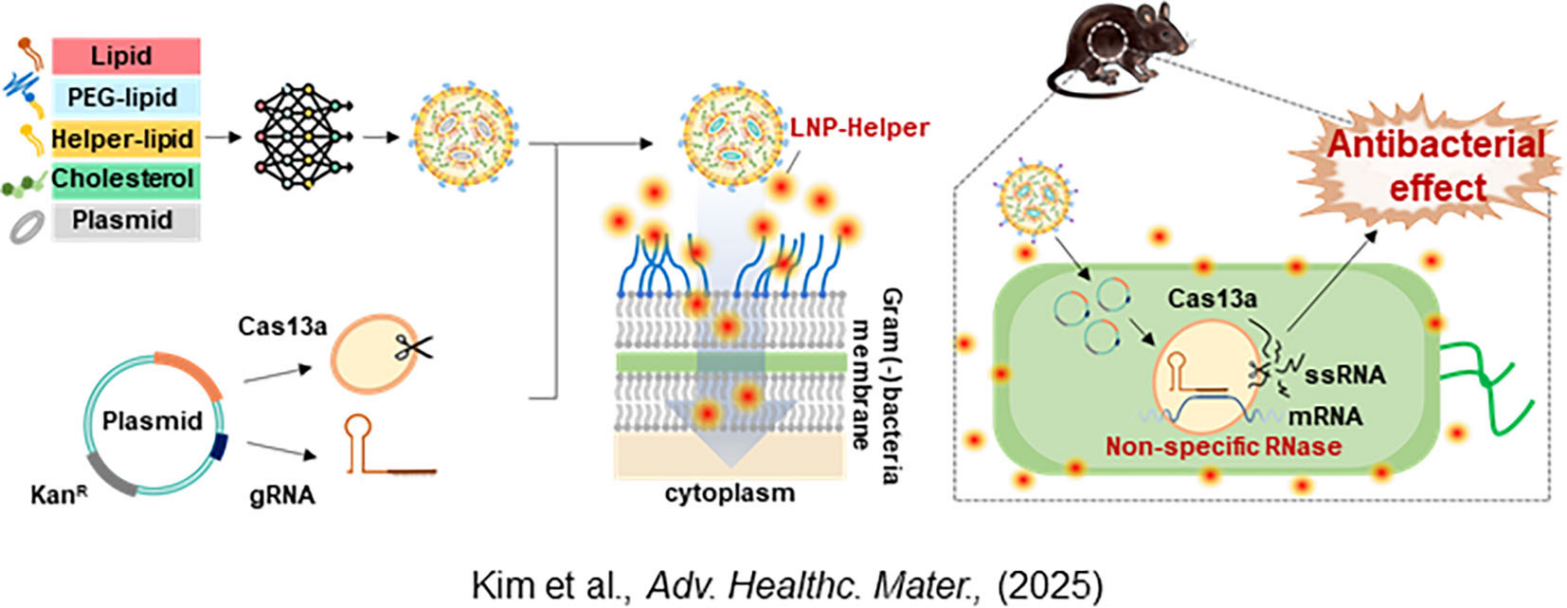
Successful delivery of CRISPR-Cas13a using LNPs in bacterial systems. LNPs were evaluated as a delivery platform for the CRISPR–Cas13a gene-editing system to combat bacterial infections. A library of 511 LNP formulations was screened, and two candidates were identified that efficiently delivered plasmids into *E. coli*, enabling the control of clinically relevant *E. coli* strains. The selected LNPs encapsulated a plasmid expressing Cas13a and a guide RNA (gRNA) targeting the 16S rRNA gene, resulting in effective bacterial cell death. Polymyxin B, a membrane-disrupting compound, was co-administered to enhance delivery efficiency by weakening the bacterial cell envelope*. In vivo* experiments using *Galleria mellonella* larvae and mouse infection models demonstrated improved survival rates, highlighting the therapeutic potential of LNP-based CRISPR delivery. This study represents a major advancement toward LNP-mediated gene-editing therapeutics for bacterial infections and offers a promising alternative to conventional antibiotics.

Recently, we provided valuable insights into the concept of LNP-helpers (Kim et al., 2025). The double outer membrane of Gram-negative bacteria appeared to be structurally incompatible with the direct application of LNPs (**Figure 1d**). However, by introducing the concept of LNP-helpers, these structural limitations could potentially be overcome (**Figure 5**). As LNP-helpers, antibiotics targeting the outer membrane such as polymyxin B and E were selected and facilitated the cytosolic entry of LNPs when applied at sub-MIC concentrations (Kim et al., 2025). The enhancement of LNP delivery efficiency observed with polymyxins implies that the double membrane of Gram-negative bacteria may constitute a critical barrier to LNP-mediated delivery. These results lead to the hypothesis that antibiotics targeting bacterial membranes may serve as potential LNP-helpers. While several helper substances have been identified for Gram-negative species (Kim et al., 2025), no such compounds have yet been reported for Gram-positive bacteria. Based on structural characteristics, the thick peptidoglycan layer of Gram-positive bacteria likely acts as a major physical barrier to LNPs (**Figure 1d**). Therefore, antibiotics that specifically target peptidoglycan are expected to serve as potential LNP-helpers for Gram-positive bacteria (Grishin et al., 2020; Sabnis and Edwards, 2023). Thus far, LNP-helpers have been co-administered with LNPs to enable nucleic acid delivery. However, if these helper molecules were conjugated directly to the PEG–lipid component of LNPs and used as target moieties, it may be possible to achieve autonomous bacterial gene delivery using LNPs alone.

Taken together, current findings indicate that the development of bacteria-targeting LNPs is fundamentally constrained by the unique physical and chemical architecture of bacterial membranes, including the LPS-rich outer membrane of Gram-negative species and the thick peptidoglycan layer of Gram-positive bacteria. Despite recent progress, the molecular mechanisms governing LNP–bacterial membrane interactions remain poorly understood, largely because existing LNPs were originally optimized for eukaryotic systems. The strategies discussed in this section—such as modifying lipid structure, tuning physicochemical parameters, and incorporating helper molecules—can be viewed as complementary approaches aimed at overcoming distinct aspects of these barriers. Moving forward, a generalized design principle is likely to emerge from integrating bacterial membrane–specific lipid engineering, molecular dynamics–based prediction tools, and co-administration strategies that transiently weaken membrane integrity. Such a framework may accelerate the rational development of LNP formulations capable of efficient and reproducible nucleic acid delivery across diverse bacterial taxa.

## 5 Production methods of LNPs

The components used to encapsulate nucleic acids in LNPs include ionizable lipids, helper lipids, cholesterol, PEG–lipids, and targeting moieties (**Figure 1b**). Ionizable lipids electrostatically self-assemble with negatively charged nucleic acids under acidic conditions, forming the core structure of LNPs (Chaudhary et al., 2021). This self-assembly process occurs almost instantaneously within microfluidic mixing systems. In typical formulations, lipids and cholesterol are dissolved in ethanol, whereas RNA or DNA intended for bacterial delivery is dispersed in an acidic aqueous phase (**Figure 1b**). When these two fractions are combined using microfluidic mixing, spontaneous self-assembly takes place, leading to the rapid formation of LNPs (Shepherd et al., 2021). Finally, targeting moieties are conjugated to the LNP surface to enable specific interactions with bacterial cells (Chaudhary et al., 2021).

### 5.1 Microfluidics and rheology analysis required for LNP production

Rheological principles have also proven highly useful in the biological sciences, such as in studying red blood cell dynamics in blood vessels, cholesterol deposition, and disease diagnostics based on blood flow properties. More recently, rheology (**Box 1**) has been applied to the decoding of DNA containing bioinformation and in genetic manipulation at the molecular level (Schowalter, 1993; Barnes, 2002; Hu et al., 2011).

Box 1 **Brief introduction of Rheology.**

The term *rheology* derives from the Greek words *rheo* (“flow”) and *logia* (“the study of”). This field of science originated in the 1920s, built upon the concept that “everything flows”—that is, all materials, including plastics, oils, foods, pharmaceuticals, batteries, cosmetics, and biomaterials, undergo flow and deformation. Since nearly all mass-produced products are formed through such processes, understanding and predicting material flow and deformation are of fundamental importance.

**Figure 6:**
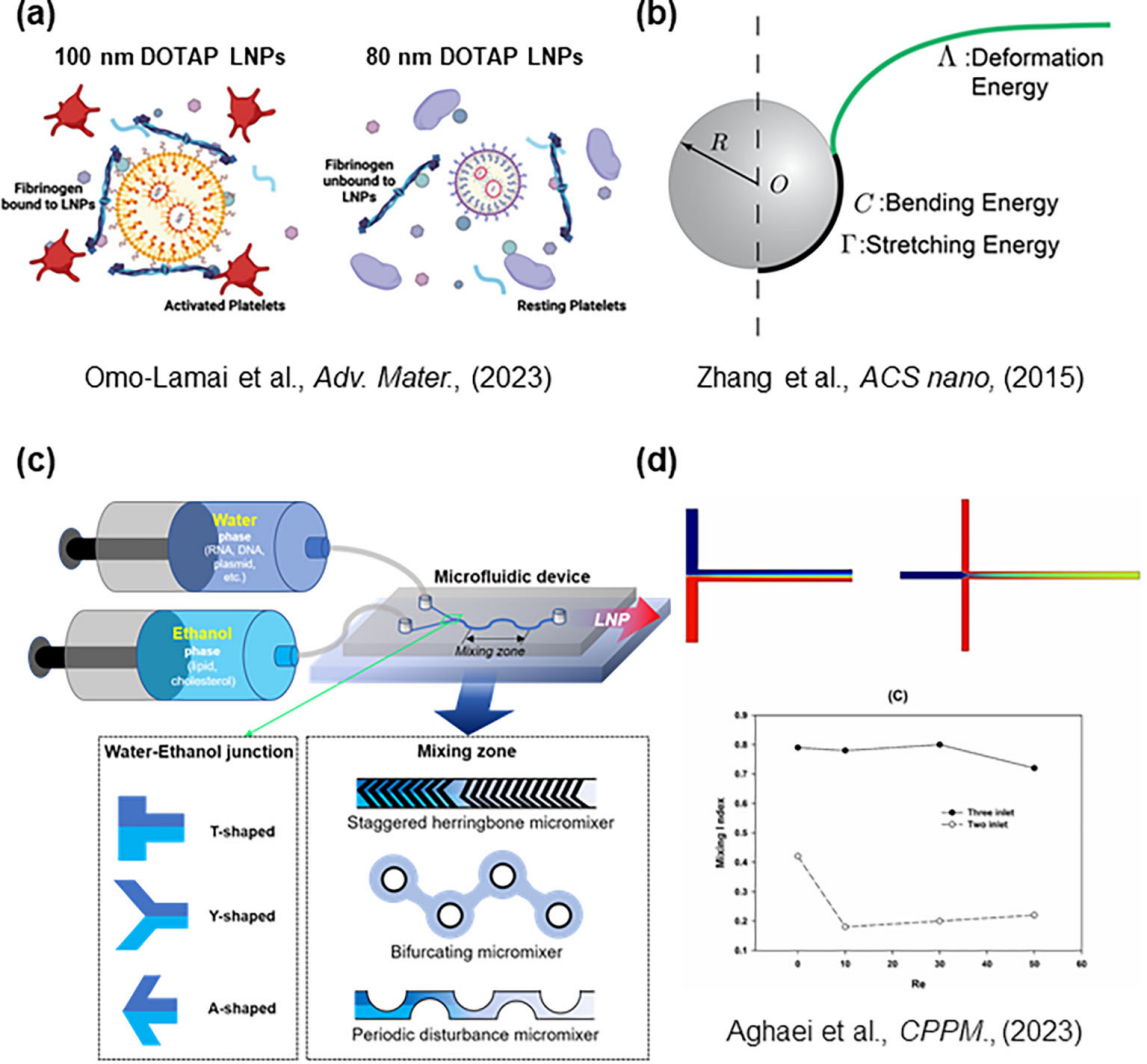
Rheological and microfluidic parameters influencing LNP production. **(a)** LNPs containing cationic lipids such as DOTAP tend to aggregate with fibrinogen; reducing particle size mitigates this effect. **(b)** Theoretical prediction of cellular uptake efficiency based on the Péclet number (Pe) and the incident angle of nanoparticles on the cell membrane. Uptake efficiency varies as a function of these physical parameters. **(c)** Schematic representation of LNP formation through microfluidic mixing of aqueous and ethanol phases. Key design features determining mixing efficiency include the junction (where the two fluids meet) and the mixing zone (where complete blending occurs). **(d)** Example of junction zone simulation performed using COMSOL Multiphysics^®^ software to visualize the mixing dynamics of the two fluid streams.

The first LNPs developed for bacterial delivery employed cationic lipids (Kim et al., 2025); nevertheless, these formulations may pose toxicity risks due to their interactions with serum proteins (Omo-Lamai et al., 2024). However, more recent studies have shown that reducing the particle size of LNPs can mitigate these adverse effects (**Figure 6a**). These observations highlight the importance of minimizing LNP size, which must be carefully considered during the manufacturing process. In particular, rheological factors play a significant role in determining nanoparticle formation dynamics. From a physical standpoint, the size of LNPs is a crucial parameter influencing biological interactions, as all biological activity inherently involves the flow and deformation of matter. Understanding the physical interplay between fluid flow and nanoparticles is therefore essential. This relationship can be quantitatively described using the Péclet number (Pe =  Ur/D, where *U* is the velocity of a particle under external force, *r* is the particle radius, and *D* is the diffusion coefficient). Based on this principle, theoretical models were developed to predict changes in nanoparticle uptake efficiency as a function of particle size (**Figure 6b**) (Chen et al., 2020). According to these models, size regulation is the dominant factor influencing cellular uptake, underscoring its critical role in LNP design. Microfluidics has emerged as a key technique for controlling LNP size (**Figure 6c**). The theoretical framework underlying this process is based on the population balance equation (PBE) theory (**Table 2**) (Ramkrishna, 2000). PBE theory indicates that the key parameter governing size control is the mixing time between the aqueous and ethanol phases (**Figure 6c**). Practically, this can be modulated by adjusting the flow rate and temperature during microfluidic mixing. The self-assembly of lipids and nucleic acids occurs through hydrodynamic mixing within microfluidic channels. Two major design features determine the mixing efficiency: one is the junction where the aqueous and ethanol phases first meet and the other is the mixing zone where complete blending occurs (**Figure 6c**). Earlier studies primarily focused on how the geometry of the mixing zone affected LNP formation efficiency and particle size (Shepherd et al., 2021). However, this review emphasizes the significance of the initiation point, or *junction*, where the two phases converge. Previous research demonstrated that efficient mixing at this initial interface is crucial for uniform nanoparticle formation (Maeki et al., 2015). The geometry of the junction is typically categorized into A-, T-, and Y-type configurations based on the angle at which the aqueous and ethanol streams intersect (**Figure 6c**) (You et al., 2017). Upon careful analysis of junction behavior, it becomes evident that optimization should proceed in a stepwise manner, beginning with the junction design before refining the downstream mixing zone.

**Table 2:** Population balance equation (PBE).

*The design of lipid nanoparticle (LNP) production devices can be fundamentally approached using the Population Balance Equation (PBE), which mathematically describes the dynamics of particle size distributions in a system. PBE is essential for understanding how different factors, such as lipid composition and process conditions, influence the formation and stability of nanoparticles. By applying PBE, researchers can predict the size distribution of LNPs, optimize production parameters to achieve desired characteristics, and ensure scalability from laboratory to industrial applications. This approach allows for better control over the encapsulation efficiency and delivery efficacy of LNPs, making PBE a valuable tool in enhancing the performance of drug delivery systems.	
$\frac{\partial n\left(V,t\right)}{\partial t}=G\left(V,t\right)+\nabla \left(\frac{D\left(V,t\right)\partial n\left(V,t\right)}{\partial V}\right)+S\left(V,t\right)$	
**Nucleation rate** $G\left(V,t\right)=k_{n}{\left[c\right]}^{n}e(-V/V_{c}$)	* Factors to consider for controlling the size of lipid nanoparticles (Conditions for making nanoparticles small) n(V,t): particle density function at particle size *V* and time *t* Lipid concentration in solution ( [c]): High [c] increases nucleation rate. Nucleation rate constant ( $k_{n}$): A higher $k_{n}\;$ increases the nucleation rate. Nucleation reaction order (n): n represents the number of lipid molecules participating in the nucleation reaction. Critical particle size ( $V_{c}$): Only particles of size $V_{c}$ or larger can participate in the nucleation process. Particle size (*V*): Small particles diffuse faster than larger particles. Fluid viscosity (*μ*): Low *μ* facilitates particle diffusion. Absolute temperature (*T*): High T increases particle diffusion. Particle aggregation rate constant ( $k_{a}$): Higher $k_{a}$ increases particle extinction rate.
**Particle diffusion coefficient** $D\left(V,t\right)=k_{d}T/\mu$	
**Particle extinction rate** $S\left(V,t\right)=-k_{a}n\left(V,t\right)$	

### 5.2 Junction design of microfluidics

Simulation studies on junction design suggest that the point at which two fluid streams converge can be used to evaluate mixing efficiency (**Figure 6d**) (Orsi et al., 2013; Shinde et al., 2021; Aghaei and Solaimany Nazar, 2023). In simulations of T-shaped junctions, a sharp increase in mixing efficiency is predicted when the Reynolds number (Re; the ratio of fluid inertia to viscous forces) reaches approximately ≥ 230 (Orsi et al., 2013). Commercial finite element software such as COMSOL Multiphysics^®^ (COMSOL, Sweden) can be employed to analyze the flow and mixing behavior of two fluids under varying operating and geometric conditions. Similarly, ANSYS Fluent (ANSYS Inc., USA) provides a robust computational fluid dynamics (CFD) platform for simulating microfluidic mixing processes (Orsi et al., 2013; López et al., 2021; Shinde et al., 2021; Aghaei and Solaimany Nazar, 2023).

Another critical consideration in microfluidic-based LNP production is the requirement for high pressure under high flow rate conditions. At elevated pressures, variations in flow stabilization time can lead to inconsistencies in product quality. This challenge arises because maintaining stable flow control becomes increasingly difficult as pressure drop increases (You et al., 2015; Hong et al., 2021). The issue is particularly pronounced at the laboratory scale, where microchannels are often fabricated from polydimethylsiloxane (PDMS), a soft elastomeric material. The swelling of PDMS structures can distort channel geometry and compromise precise flow regulation (Dangla et al., 2010). As a result, magnetic motor–driven syringe pumps often fail to maintain a consistent flow rate under such conditions. For more stable and reproducible LNP production, pressure-controlled syringe pumps are therefore recommended.

In summary, the production of LNPs is governed by a set of fundamental physical parameters—namely mixing time, flow regime, and material stability—that collectively determine particle size, uniformity, and reproducibility. Although various microfluidic designs and operational conditions have been described, these approaches ultimately converge on the shared objective of achieving precise and scalable control over LNP self-assembly. As the field advances, integrating population balance modeling, rheology-based predictions, and optimized junction engineering will be essential for establishing standardized manufacturing principles. Moreover, the incorporation of pressure-stable microfluidic platforms and computational tools for real-time process optimization may further enhance the reliability of LNP production, facilitating the translation of nucleotide-based antimicrobial formulations into practical applications.

## 6 Concluding remarks and future perspectives

This review highlights the emerging concept of nucleotide-based antibiotics delivered by lipid nanoparticles, a strategy that integrates programmable gene-targeting therapeutics with a clinically validated delivery platform. However, their successful commercialization critically depends on the optimization of efficient delivery systems capable of overcoming the unique structural and physicochemical barriers of bacterial cells. While we have demonstrated the feasibility of using LNPs for delivering nucleic acids into bacteria (Kim et al., 2025), several unresolved challenges hinder broader application. These include the limited understanding of LNP–bacterial membrane interactions, the absence of bacterial membrane–specific lipid design principles, variability in delivery efficiency across species, and the need for scalable manufacturing processes that ensure consistent particle properties suitable for *in vivo* use.

Future progress will require a mechanistic framework explaining how LNPs traverse bacterial outer membranes, peptidoglycan layers, and cytoplasmic membranes. Integrating molecular dynamics simulations, lipid–membrane interaction assays, and high-resolution structural analyses may accelerate the discovery of lipid motifs with enhanced fusogenicity tailored to bacterial systems. In parallel, constructing bacteria-specific lipid libraries informed by the chemical diversity of bacterial envelope components—such as lipid A or teichoic acids—could provide a foundation for rational LNP design. Strategies incorporating targeting moieties or helper molecules may further improve delivery efficiency, particularly for species with robust outer membranes or thick cell walls.

From an engineering perspective, advances in microfluidic manufacturing and rheology-informed process control will be essential for producing LNPs with the particle sizes, homogeneity, and stability required for therapeutic deployment. Simulation-based mixing optimization, automated pressure stabilization, and high-throughput formulation screening may further support scalable and reproducible production. As these technologies evolve, comprehensive *in vivo* evaluations—including toxicity, biodistribution, pharmacokinetics, and microbiome impact—will be critical for safe clinical application. In addition, evaluating long-term safety including potential immunogenicity, lipid accumulation, and microbiome alterations will be essential, particularly for repeated or chronic administration.

Looking ahead, nucleotide-LNP technologies are expected to extend far beyond antimicrobial applications. CRISPR-based tools was used to modulate gut microbiota and enhance the beneficial functions of commensal bacteria (Hidalgo-Cantabrana et al., 2017). Building upon these advances, LNPs carrying engineered CRISPR–Cas systems could be designed not only to suppress pathogenic traits but also to fine-tune bacterial gene expression or endow microbes with new, host-protective functionalities. Such approaches may enable precise reprogramming of microbial communities in ways that complement traditional antimicrobial therapies.

Furthermore, clinical translation of nucleotide-based antibiotics is anticipated to benefit from programmable sequence design and predictive computational analyses. With the rapid expansion of genomic and metagenomic datasets for host and microbial species, off-target interactions can increasingly be forecasted and minimized through in silico optimization. These developments position LNP-mediated nucleic acid delivery as a transformative platform—not only for treating antibiotic-resistant infections, but also for engineering microbial ecosystems to promote long-term human health.
